# The Efficacy of Denosumab in the Management of a Tibial Paediatric Aneurysmal Bone Cyst Compromised by Rebound Hypercalcaemia

**DOI:** 10.1155/2020/8854441

**Published:** 2020-12-09

**Authors:** Matthew Harcus, Samantha Aldridge, Adesegun Abudu, Lee Jeys, Senthil Senniappan, Henry Morgan, Barry Pizer

**Affiliations:** ^1^Department of Paediatric Oncology, Alder Hey NHS Foundation Trust, Liverpool, UK; ^2^Department of Paediatric Nephrology, Alder Hey NHS Foundation Trust, Liverpool, UK; ^3^Department of Orthopaedic Oncology, The Royal Orthopaedic Hospital, Birmingham, UK; ^4^Department of Paediatric Endocrinology, Alder Hey NHS Foundation Trust, Liverpool, UK

## Abstract

Surgery is the main treatment option for patients with aneurysmal bone cyst (ABC). We report our experience of using denosumab as an alternative treatment in a child with a multiply recurrent and unresectable tibial ABC. The efficacy and safety of denosumab in the paediatric population, and in the treatment of ABC, are still to be fully evaluated. We describe a 13-year-old boy with an extensive and aggressive ABC involving the proximal tibia, which had recurred following multiple previous surgeries. The patient had ongoing severe pain, was unable to weight-bear, and was at significant risk of pathological fracture. En bloc resection and embolization were not deemed viable, and a decision to use denosumab was made. He received 17 doses of subcutaneous denosumab (70 mg/m^2^) over a 27-month period, at increasing dose intervals. His symptoms significantly improved, and bony consolidation was observed within six months of treatment. He was able to walk without protection and fully weight-bear without any pain by 18 months. With an increase to a six-month dosing interval, the patient presented with a severe, symptomatic rebound hypercalcaemia requiring bisphosphonate therapy. This reoccurred on two further occasions. This case adds to the evidence that denosumab is effective in the treatment of ABC in paediatric patients, but there is a risk of rebound hypercalcaemia. Therefore, patient awareness and biochemical monitoring for rebound hypercalcaemia are essential.

## 1. Introduction

Aneurysmal bone cysts (ABCs) are benign but locally destructive osteolytic lesions. They typically present with pain, swelling, and sometimes pathological fractures. They are most commonly diagnosed in the first two decades of life, with 70% being primary lesions and the remaining 30% secondary to other pathology [1–4].

Management of ABCs is challenging with risks of significant morbidity, particularly with spinal lesions, and a high rate of recurrence [5, 6]. Surgery is the mainstay of treatment, including conservative surgery such as curopsy or curettage, which are associated with less morbidity but a higher risk of recurrence compared to en bloc resection [7–9]. Curettage may be used with local adjuvant therapies, such as sclerotherapy. Larger or unresectable lesions may be treated by selective arterial embolization only, or in combination with surgery [7]. The role of medical management is less well described.

Denosumab is a human monoclonal antibody that binds to the cytokine receptor activator of nuclear factor-kappa B ligand (RANKL). RANKL activates the RANK receptor of osteoclasts, leading to bone resorption, with denosumab inhibiting this interaction [10]. The RANKL-RANK interaction has been implicated in the pathogenesis of giant cell tumour of the bone, another destructive bone tumour, with osteoclast-like giant cells highly expressing RANK [11, 12]. A number of studies have demonstrated the efficacy of denosumab in the treatment of giant cell tumour of the bone [13–22]. Given the similarly high expression of RANK in the giant cells of ABC, alongside the high expression of RANKL in neoplastic stromal cells, the potential for denosumab therapy has been extended to ABCs [23]. An increasing number of reports have demonstrated a beneficial effect with the use of denosumab therapy in paediatric patients with these lesions [23–33].

Complications of denosumab therapy include on-treatment hypocalcaemia, hypophosphataemia, osteonecrosis of the jaw, osteosclerosis, and atypical fractures [21, 34, 35]. A less well-described adverse event is a rebound hypercalcaemia seen following cessation of denosumab treatment. This has been reported in four patients with ABCs following denosumab therapy [31–33].

Here, we describe a case, in a paediatric patient, demonstrating the effectiveness of denosumab in the treatment of a tibial ABC, complicated with severe rebound hypercalcaemia on increasing the dosing interval to six months.

## 2. Case Description

**Figure 1:**
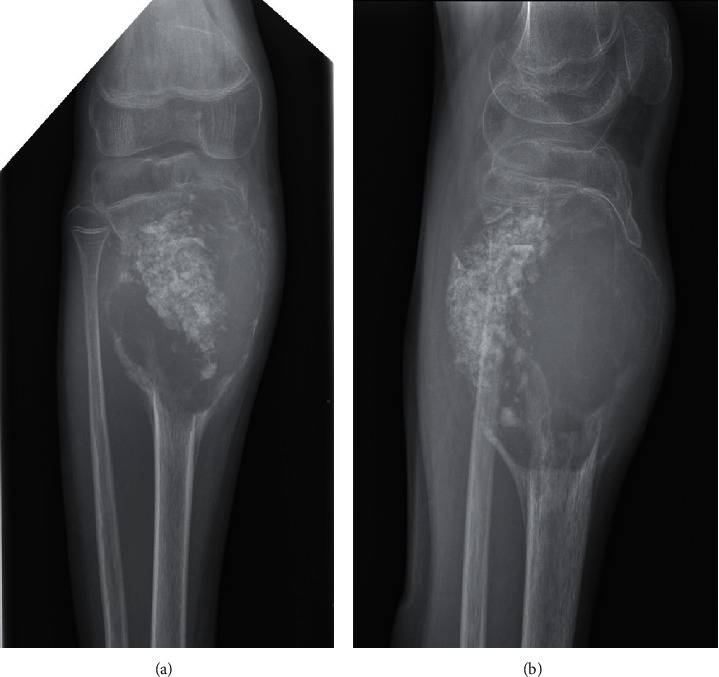
Anteroposterior (a) and lateral (b) plain radiographs of the right lower leg demonstrating a recurrent ABC after initial detailed curettage and allogenic bone grafting.

**Figure 2:**
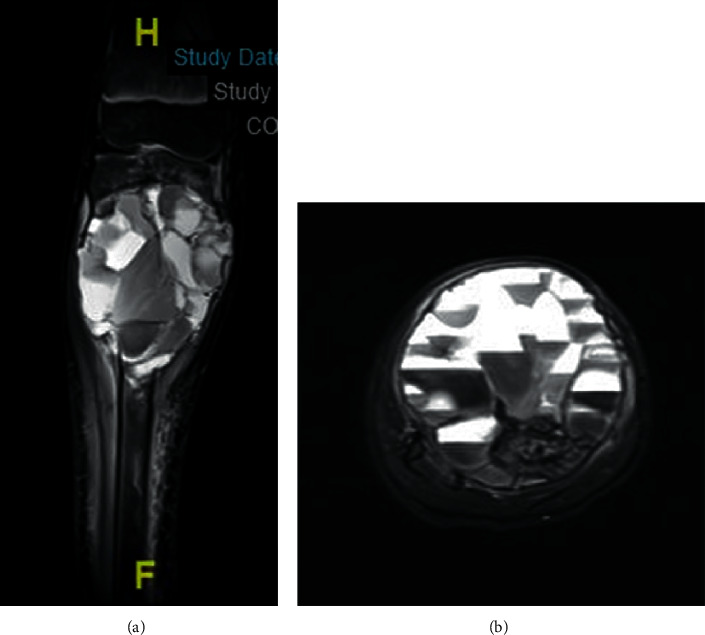
Coronal (a) and axial (b) T2 weighted MRI images of the tibia showing a large recurrent ABC.

A 13-year-old, previously fit and well, male presented with pain and swelling to his right lower leg. A bone cyst was identified on imaging including plain radiographs and MRI scans (Figures 1 and 2). He underwent curettage on three occasions, including one with a bone graft, with recurrence on each occasion. An ABC was confirmed on biopsy on tissue obtained during curettage. En bloc resection of the proximal tibia was not thought to be viable due to the extent of disease and involvement of major neurovascular structures. Vascular embolization was not possible because no feeding vessels were identified on angiogram. Throughout this period, he had ongoing severe leg pain, was unable to weight-bear or attend school, and was at significant risk of pathological fracture.

Denosumab therapy was subsequently commenced following discussion of a bone tumour treatment multidisciplinary team. The weight at the start of treatment was 36 kg, with a height of 161 cm. The patient received four doses of subcutaneous denosumab (70 mg/m^2^) on a weekly basis for four weeks with significant improvement in pain, allowing him to return to school shortly after, although he was still immobile. There was a subtle initial radiological response with a slight reduction in size of the lesion. There was asymptomatic mild hypocalcaemia (corrected calcium 2.03 mmol/L; normal range 2.25–2.74 mmol/L) in the early stages of treatment which was treated with oral calcium supplementation (calcium carbonate 1.25 g daily). He remained on this supplementation throughout treatment. The patient went on to receive six further doses of denosumab at four-week intervals at the same dose.

**Figure 3:**
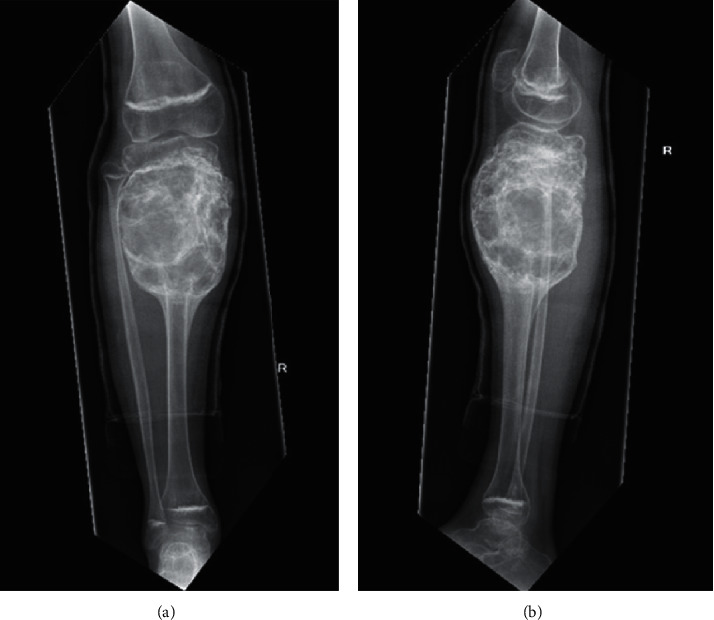
Anteroposterior (a) and lateral (b) plain radiographs of the right lower leg following six months of denosumab therapy, demonstrating significant interval change in the texture of the cyst, with evidence of new bone formation. Calcification of growth plates was noted which led to the decision to begin increasing the dose interval.

On review following six months of treatment, there was ongoing radiological improvement, with bony consolidation (Figure 3). The patient remained pain free, and he began partial weight-bearing over the subsequent few months. However, there was also evidence of some calcification of the lower limb growth plates, possibly as a result of denosumab therapy. Therefore, the dose interval was increased initially to two months, and then at 12 months of treatment, to three months.

After 18 months of treatment, the patient was walking without protection and fully weight-bearing without pain. The ABC was stable radiologically, and the dosing interval was further increased to four months.

On review after 27 months of treatment (following the 17^th^ dose; cumulative dose 1554 mg), the patient remained pain free and was able to play gentle sport. Given the good response, the plan was for two further doses of denosumab every six months before stopping therapy.

On presentation for the first six-month dose, the patient was found to have a blood pressure of 170/100 mmHg. He reported he had felt generally unwell for several weeks with malaise, anorexia, polyuria, and headaches. His weight was 47 kg which was significantly lower than his last clinic review four months earlier when it was 50.7 kg, demonstrating a weight loss of 3.7 kg. Height was static in this four-month period at 172 cm. Initial blood tests demonstrated hypercalcaemia (corrected calcium 4.04 mmol/L). There were normal levels of phosphate (1.35 mmol/L; normal range 0.74–1.55 mmol/L) and alkaline phosphatase (201*µ*/L; normal range 55–236*µ*/L). 25-Hydroxyvitamin D2 was <5 nmol/L, and 25-hydroxyvitamin D3 was 84 nmol/L (normal range >50 nmol/L). In addition, there was an acute kidney injury (AKI) with a creatinine of 293 *µ*mol/L (normal range 46–102 *µ*mol/L) and a urea of 13.4 mmol/L (normal range 2.5–6.7 mmol/L). His parathyroid hormone level on presentation was suppressed at 1.0 pmol/L (normal range 1.1–6.9 pmol/L). Urine calcium/creatinine ratio was high at 3.62 mm/mm Cr (normal range 0–0.6 mm/mm Cr), however was not performed until 11 days after presentation and initial management. Lab values on this presentation and in subsequent management are summarised in Table 1.

Initial management was with intravenous (IV) fluids, IV furosemide (60 mg six hourly), and cessation of the supplemental calcium. There was no initial biochemical improvement, with corrected calcium peaking at 4.18 mmol/L and creatinine at 314 *µ*mol/L. 200 units of calcitonin, initially subcutaneously, and then intravenously, was introduced once a day with limited improvement in calcium level and renal function, despite increasing to twice daily dosing over a nine-day period. Therefore, two doses of IV pamidronate (0.25 mg/kg and then 0.5 mg/kg, 24 hours apart) were given. Bisphosphonate therapy led to the normalisation of calcium levels (corrected calcium 2.74 mmol/L) within three days, with subsequent improvement to normal range of the high creatinine (97 *µ*mol/L) a further three days later. Amlodipine (2.5 mg daily), which had been commenced to control the hypertension, could also be stopped at this stage with no adverse effect on the now normal blood pressure. He was discharged the following day.

Calcium levels were monitored closely following discharge with a further episode of rebound hypercalcaemia (corrected calcium 3.44 mmol/L), with an associated AKI (creatinine 115 *µ*mol/L), 13 days after initial pamidronate treatment (Table 1). A further dose of IV pamidronate (0.5 mg/kg) was given, again with a positive response (corrected calcium 2.50 mmol/L four days later). A further episode occurred two weeks later (corrected calcium, 3.15 mmol/L), this time successfully treated with IV zoledronic acid (0.05 mg/kg).

A bone density scan (DEXA scan) was performed approximately two months following the third episode of hypercalcaemia and was within normal range, with a *Z*-score of 0.5 for the lumbar spine and 1.0 for the whole body (normal range >−2.0). Recurrence of hypercalcaemia has not subsequently occurred, and the patient remains mobile and pain free 16 months since the last denosumab dose and nine months since the third and last episode of hypercalcaemia. Now at 18 years old, his fatigue and appetite, without the need for a low calcium diet, have improved and he is gaining weight (now 53.8 kg).

## 3. Discussion

In this case report, we describe the second reported successful use of denosumab, and first in a child, with an ABC involving the tibia. There was a rapid clinical response within just four weeks of commencing treatment, with a more apparent radiological response within six months. This has been sustained, at the time of writing, 16 months after the last dose. This response to RANKL inhibition is consistent to other paediatric cases previously described and highlights the potential role of denosumab in these locally aggressive and destructive lesions [23–33].

Whilst the beneficial effects of denosumab in treating paediatric cases of ABCs are becoming better defined, the potential adverse effects still remain unclear, with problems in calcium homeostasis potentially significant. On-treatment hypocalcaemia has been well described in denosumab therapy, and occurred in this case, being asymptomatic and managed relatively simply with additional calcium supplementation [15]. Rebound hypercalcaemia, although rarer and less well described, is as a potential complication of treatment cessation, or in this case, increasing the dose interval to six months [31–33].

The cause for rebound hypercalcaemia is thought to be related to osteoclast overactivity after stopping treatment with denosumab. High bone turnover in skeletally immature children may increase this risk, potentially accounting for the increasingly reported cases of this complication as denosumab use in younger patients becomes more frequent [35]. The majority of reported cases of rebound hypercalcaemia related to denosumab have occurred in children or teenagers, with the youngest aged eight years old [34]. Reports involving adults are less common, with the oldest occurring in a 67-year-old patient who had been on denosumab to treat osteoporosis for ten years [36].

The described cases of, often severe, rebound hypercalcaemia highlight the risks of sudden treatment cessation and therefore the need to wean off denosumab therapy. Furthermore, appropriate monitoring is required following cessation and, as demonstrated in this case, during the weaning process itself. There are currently no guidelines for the appropriate weaning or monitoring of patients being treated with denosumab for ABCs. Whilst the half-life of denosumab following treatment in adult patients has been estimated at 29 days (range 25–35 days), this does not take into account accumulated doses and may not be extrapolated to the paediatric population [35, 37]. In this case, hypercalcaemia was identified six months after the previous dose of denosumab, although given biochemical monitoring did not occur in this period it is difficult to estimate its actual onset. Previously reported episodes of rebound hypercalcaemia have occurred as soon as seven weeks, and as long as seven months, after the last dose [34, 35]. Of note, the case arising seven weeks after the last dose of denosumab was just four days after a normal serum calcium level was measured [34]. This variation, in addition to a potentially sudden rise in calcium level, demonstrates the difficulties in observing for this potential adverse event. Our centre now recommends biochemical monitoring at least every two months during, and for at least six months following, treatment with denosumab in this setting, in addition to the education of patients and their families on the symptoms of hypercalcaemia. The problem is likely to be compounded by concomitant use of calcium supplements due to the on-treatment risk of hypocalcaemia, as in this case; thus, the need for these should also be reviewed regularly.

## 4. Conclusion

Whilst surgical options remain the mainstay of ABC management, there remains significant risk of associated morbidity and a high recurrence rate. This case report adds to the growing evidence for the beneficial effects of the use of denosumab as an adjuvant therapy in these lesions.

With increased use in a younger population of patients, however, the side effects of denosumab therapy in this scenario are becoming more apparent. In addition to the more established risk of hypocalcaemia, patients on denosumab treatment should also be informed of the potential risk of hypercalcaemia on-treatment cessation, or on increasing the dosing interval. They should be made aware of potential symptoms in addition to undergoing regular monitoring of serum calcium levels at least every two months.

## Figures and Tables

**Table 1:** Summary of key lab values that were available at initial, and subsequent, presentations with hypercalcaemia, with levels pre- and post-bisphosphonate treatment.

	Initial presentation	Pre-1^st^ treatment	Post-1^st^ treatment	2^nd^ presentation	Post-2^nd^ treatment	3^rd^ presentation	Post-3^rd^ treatment
Corrected calcium (mmol/L)	4.04	3.53	2.74	3.44	2.50	3.15	2.35
Phosphate (mmol/L)	1.35	1.22	0.70	1.47	0.75	1.33	1.03
ALP (*µ*/L)	201	204	193	198	158	151	135
Creatinine (*µ*mol/L)	293	179	172	115	99	82	99
PTH (pmol/L)	1.0					<0.3	2.7

Normal ranges: corrected calcium, 2.25–2.74 mmol/L; phosphate, 0.74–1.55 mmol/L; alkaline phosphatase (ALP), 55–236 *µ*/L; creatinine, 46–102 *µ*mol/L; and parathyroid hormone (PTH), 1.1–6.9 pmol/L.

## Data Availability

No data were used to support this study.
